# Comparison of an In‐House Multiplex Real‐Time PCR Method With Altona Diagnostics Kits in the Detection of HSV, VZV, and EBV Viruses in Transplant Patients

**DOI:** 10.1155/bmri/7109372

**Published:** 2025-09-22

**Authors:** Reyhaneh Kalhor, Mahdi Paryan, Sirous Naeimi, Hourieh Kalhor, Hassan Noorbazargan, Samira Mohammadi-Yeganeh

**Affiliations:** ^1^ Department of Genetics, College of Science, Kazerun Branch, Islamic Azad University, Kazerun, Iran, azad.ac.ir; ^2^ Department of Research and Development, Production and Research Complex, Pasteur Institute of Iran, Tehran, Iran, pasteur.ac.ir; ^3^ Cellular and Molecular Research Center, Qom University of Medical Sciences, Qom, Iran, muq.ac.ir; ^4^ Roje Technologies Center, Tehran, Iran; ^5^ Medical Nanotechnology and Tissue Engineering Research Center, Shahid Beheshti University of Medical Sciences, Tehran, Iran, sbmu.ac.ir; ^6^ Department of Molecular Medicine, School of Advanced Technologies in Medicine, Shahid Beheshti University of Medical Sciences, Tehran, Iran, sbmu.ac.ir

**Keywords:** EBV, HSV, in-house assay, multiplex real-time PCR, transplant patients, VZV

## Abstract

**Background and Objectives:** Herpes simplex virus (HSV), varicella‐zoster virus (VZV), and Epstein–Barr virus (EBV) infections pose significant challenges in managing transplant patients and necessitate rapid and precise diagnostic methods due to their immunosuppressed state. This study designed and evaluated the performance of an in‐house multiplex real‐time PCR for simultaneous detection of these viruses.

**Materials and Methods:** Plasma samples from 270 transplant patients were tested using an in‐house multiplex real‐time PCR assay specifically designed for HSV, VZV, and EBV. Analytical specificity and the assay’s limit of detection (LOD) were determined. Statistical analyses were performed to evaluate the agreement between the in‐house assay and the reference kit.

**Results:** The method had a specificity of 98% for HSV, 97% for VZV, and 95% for EBV, alongside 100% sensitivity for all three viruses. No cross‐reactivity was observed with other viral or bacterial DNA. The LOD for the in‐house assay was determined to be 6.25, 25, and 25 copies/mL for HSV, VZV, and EBV, respectively. Additionally, precision analysis showed low CV values in both intra‐assay and interassay evaluations (HSV: 1.5%–1.8%; VZV: 2.3%–2.6%; and EBV: 3.7%–3.9%), confirming the assay’s robust analytical precision. Bland–Altman analysis showed mean differences of 1.35, −3.29, and 1.75 for HSV, VZV, and EBV, respectively. This multiplex real‐time PCR method enables detection at lower concentrations. Cross‐reactivity testing confirmed no interaction with DNA from other viruses or nontarget microorganisms. Bland–Altman and linear regression analyses also showed a strong agreement between commercial and in‐house methods.

**Conclusion:** These findings, compared to Altona diagnostic kits, highlight the value of designing and applying advanced diagnostic assays in managing viral infections in transplant patients.

## 1. Introduction

Infections caused by herpes simplex virus (HSV), varicella‐zoster virus (VZV), and Epstein–Barr virus (EBV) represent critical challenges in the clinical management of transplant patients. Due to immunosuppression, these patients are highly susceptible to opportunistic infections, which can rapidly rise to severe and life‐threatening complications [1, 2]. HSV can cause severe central nervous system, skin, and mucosal infections, often progressing to chronic and disseminated diseases in immunocompromised individuals [3]. Similarly, VZV, the causative agent of chickenpox and shingles, may result in severe complications such as pneumonitis and hepatitis in transplant recipients [4]. EBV is known for its association with chronic infections and malignancies, including Burkitt’s lymphoma and nasopharyngeal carcinoma, posing a significant risk to these patients [5].

Rapid and precise detection of these viruses is essential for effective management and prevention of life‐threatening complications. While traditional diagnostic methods such as viral culture and serology, though still in use, are limited by low sensitivity and time‐consuming procedures [6, 7], molecular‐based methods such as real‐time PCR have emerged as the gold standard for robust and accurate detection of viral infections [8, 9]. However, the high cost and technical expertise required for these methods can limit their accessibility in all clinical settings [10].

Multiplex real‐time PCR is a promising innovation in viral/microbial diagnostics, enabling the simultaneous detection of multiple organisms in a single reaction. This method not only improves diagnostic speed but also reduces costs and resource requirements [9, 11]. Importantly, it allows for the evaluation of key diagnostic parameters such as sensitivity, specificity, and analytical specificity, including cross‐reactivity with nontarget organisms, ensuring robust and reliable results. The ability to simultaneously detect HSV, VZV, and EBV in a single assay offers a unique opportunity to enhance diagnostic efficiency and accuracy, thereby preventing delays in treatment and reducing the risk of life‐threatening complications. This study is aimed at designing an in‐house multiplex real‐time PCR assay for the detection of HSV, VZV, and EBV in plasma samples of transplant patients. To validate the performance of the designed assay, its results were compared with those obtained from the commercial Altona Diagnostics RealStar PCR Kit. Key parameters such as sensitivity, specificity, analytical specificity (in silico and in vitro), and limit of detection (LOD) were evaluated. Additionally, statistical analyses, including Bland–Altman and linear regression, were performed to assess discrepancies and correlations between the two methods.

## 2. Materials and Methods

### 2.1. Clinical Samples

A total of 270 plasma samples were collected from transplant patients over 6 months from April 2024 to September 2024 at Day Hospital, Tehran, Iran. This included 50 positive and 40 negative samples for HSV and 20 positives and 70 negative samples each for VZV and EBV. All plasma samples were initially tested using the commercial Altona RealStar PCR Kits for HSV, VZV, and EBV. The results of these assays were used to classify samples as positive or negative, serving as the reference standard for this study. All samples were also tested for the presence of the human RNaseP gene to verify extraction efficiency and the absence of PCR inhibitors. To prevent DNA degradation, all samples were immediately stored at −80°C. Informed consent was received from all enrolled individuals. The study was supervised and approved by the Biomedical Ethics Committee of Iran (Ethics Code: IR.IAU.KAU.REC.1403.006).

### 2.2. Viral DNA Extraction

Viral DNA was extracted from 200 *μ*L of each plasma sample using the QIAamp DNA Mini Kit (Qiagen GmbH, Hilden, Germany), according to the manufacturer’s guidelines. Briefly, the DNA extraction procedure was performed by the addition of lysis buffer, separation of DNA with silica columns, and final elution of extracted DNA in 60 *μ*L of DNase/RNase‐free water. Positive and negative controls were included at every step to ensure data accuracy and eliminate contamination risks. In addition to positive and negative controls, each clinical sample was coamplified with an independent internal control targeting the human RNaseP gene. This control was included in a separate reaction for each sample to monitor extraction efficiency and detect potential PCR inhibition. Samples with absent or delayed RNaseP amplification (Ct > 35) were considered invalid and were re‐extracted.

### 2.3. Designing Primer Pairs and Probes

Specific primers and probes for the detection of HSV, VZV, and EBV were designed using AlleleID7 and Primer‐BLAST software. Genomic sequences for these viruses were retrieved from the NCBI database; aligned to identify conserved regions, the primers and probes were designed in conserved regions and then validated in silico using BLAST to ensure their specificity. Key parameters in primer and probe design were considered. The final sequences of primers and probes are provided in Table 1. Additionally, an internal control targeting the human RNaseP gene was included to monitor extraction efficiency and potential PCR inhibition; its primer and probe sequences are also listed in Table 1.

**Table 1:** Primer and probe sequences for HSV, VZV, EBV, and the human RNaseP internal control.

**Primer and probe names**	**Sequence**	**Length (nucleotide)**
HSV forward primer	5 ^′^ CATCACCGACCCGGAGAGGGAC 3 ^′^	22
HSV reverse primer	5 ^′^ GGGCCAGGCGCTTGTTGGTGTA 3 ^′^	22
HSV probe	FAM‐CCGCCGAACTGAGCAGACACCCGCGC‐ BHQ1	26
VZV forward primer	5 ^′^ AACTTTTACATCCAGCCTGGCG 3 ^′^	22
VZV reverse primer	5 ^′^ GAAAACCCAAACCGTTCTCGAG 3 ^′^	22
VZV probe	YakimaYellow‐TGTCTTTCACGGAGGCAAACACGT‐BHQ1	24
EBV forward primer	5 ^′^ CGGAAGCCCTCTGGACTTC 3 ^′^	19
EBV reverse primer	5 ^′^ CCCTGTTTATCCGATGGAATG 3 ^′^	21
EBV probe	ROX‐TGTACACGCACGAGAAATGCGCC‐BHQ2	23
RNase P forward primer	5 ^′^ AGATTTGGACCTGCGAGCG 3 ^′^	19
RNase P reverse primer	5 ^′^ GATAGCAACAACTGAATAGCCAAGGT 3 ^′^	26
RNase P probe	CY5‐TTCTGACCTGAAGGCTCTGCGCG‐BHQ2	23

### 2.4. Optimization of Multiplex Real‐time PCR

The multiplex real‐time PCR assay for detection of HSV, VZV, and EBV was performed using the ABI StepOne Real‐Time PCR system (Applied Biosystems, United States). The condition of multiplex reaction was optimized. Finally, the reaction mixture consisted of 10 *μ*L of QuantiTect Probe PCR Master Mix (Qiagen, Germany), 0.5 *μ*L of each primer (20 *μ*M concentration), 0.4 *μ*L of probes (10 *μ*M concentration), 5 *μ*L of extracted DNA, and required nuclease‐free water up to 20 *μ*L. Thermal cycling conditions included an initial denaturation at 95°C for 15 minutes, followed by 40 cycles of 95°C for 10 seconds and 60°C for 30 seconds. Fluorescence signals were analyzed to determine qualitative results.

### 2.5. Comparison with Reference Kits

Sensitivity, specificity, and predictive values were calculated for the In‐house multiplex real‐time PCR assay using the Altona RealStar PCR Kits as the gold standard comparator. No separate performance calculations were performed for the Altona assay, as it served exclusively as the reference method.

To compare the performance of the in‐house designed assay, reference commercial kits from Altona Diagnostics, including RealStar HSV PCR Kit 1.0 (Product Code: 061013), RealStar VZV PCR Kit 1.0 (Product Code: 071013), and RealStar EBV PCR Kit 1.0 (Product Code: 131013) were used. All reactions were conducted in exact accordance with the manufacturer’s protocols to ensure standardized experimental conditions. The results obtained from these reference kits served as the reference for evaluating the accuracy, sensitivity, and reliability of the in‐house multiplex real‐time PCR method.

### 2.6. Determination of Sensitivity, Specificity, and LOD

The performance of the In‐house assay was assessed using positive and negative samples verified by the reference kits. This included 50 positive and 40 negative samples for HSV, 20 positives and 70 negative samples each for VZV and EBV. Sensitivity was calculated as the percentage of true positive samples correctly identified, and specificity was determined as the percentage of true negative samples accurately detected.

To evaluate analytical specificity, in‐silico and in‐vitro analyses were conducted. BLAST searches against the NCBI nucleotide database were performed to ensure that the primers and probes were specific to the target sequences of HSV, VZV, and EBV, with no significant homology to genomes of related viruses, including Cytomegalovirus (CMV), Human Herpesvirus 6 (HHV‐6), Human Herpesvirus 7 (HHV‐7), and Human Herpesvirus 8 (HHV‐8). In‐vitro cross‐reactivity testing was carried out to assess the assay’s specificity using clinical DNA samples from CMV, HHV‐6, HHV‐7, HHV‐8, and bacterial species (*Staphylococcus aureus* and *Escherichia coli*). For each non‐target pathogen, nucleic acids were prepared at concentrations ≥10^6^ copies/*μ*L (for viral DNA) or ≥106 CFU/mL (for bacterial DNA), which is equal to or exceeds the highest concentrations typically observed in clinical specimens. These challenging conditions were selected to ensure that the assay maintained high specificity even in the presence of high loads of non‐target organisms.

The LOD for detection of each virus was determined using serial 10‐fold dilutions of plasmid DNA standards containing the target regions for HSV, VZV, and EBV. Plasmid concentrations were measured using a Qubit fluorometer (Thermo Fisher Scientific, United States) and converted to genome copy numbers based on the molecular weight of the plasmid and Avogadro’s constant. These defined standards were then serially diluted in nuclease‐free water to prepare concentrations ranging from 10^6^ to 1 copies/*μ*L. Each dilution was tested in 20 replicates to determine the lowest concentration detectable with ≥ 95% positivity.

### 2.7. Evaluation of Precision

The precision of the in‐house method and the reference kits for detecting HSV, VZV, and EBV was evaluated following Clinical and Laboratory Standards Institute (CLSI) guidelines (EP05‐A3). For intra‐assay precision (repeatability), each virus was tested in triplicate within a single run under identical conditions (same day, same operator, and same equipment). For interassay precision (intermediate precision), the same panel of samples was tested in three independent runs performed on three consecutive days to assess day‐to‐day variability. The test panel included 50 positive samples for HSV and 20 positive samples each for VZV and EBV. In both assessments, cycle threshold (Ct) values were recorded, and standard deviations (SDs) and coefficients of variation (CVs) were calculated. Positive and negative controls were included in each run to ensure assay validity.

### 2.8. Statistical Analysis

To evaluate the agreement between the in‐house method and the reference kits, statistical analyses were performed using Microsoft Excel 36.5. Bland–Altman analysis was used to find agreement between the in‐house method and reference kits. Differences in results were calculated, and the mean difference served as a measure of overall agreement. The 95% confidence limits (upper and lower limits of agreement) were computed separately for HSV, VZV, and EBV. Bland–Altman plots were generated to visualize agreement, with deviations outside the 95% confidence limits indicating potential discrepancies. Linear regression was used to evaluate the correlation and linear alignment between the results of the in‐house method and the reference kits. Coefficients of determination (*R* ^2^) were calculated for HSV, VZV, and EBV to quantify correlation strength, with values closer to 1 indicating higher accuracy. Scatter plots displayed data alignment between the methods. Results of the reference kits were considered the gold standard, and results of the in‐house method were compared to them to determine sensitivity, specificity, and overall performance.

## 3. Results

### 3.1. Performance of the In‐House Multiplex Real‐Time PCR Method Compared to Altona Diagnostics Reference Kits (RealStar PCR Kit)

The performance of the in‐house multiplex real‐time PCR method for the simultaneous detection of HSV, VZV, and EBV viruses was evaluated using a total of 270 clinical samples. This included 50 positive and 40 negative samples for HSV and 20 positives and 70 negative samples each for VZV and EBV. The results showed that the Iin‐house method had a sensitivity of 100% in detecting the HSV virus and correctly identified all 50 positive samples. Among the HSV negative samples, the kit had a specificity of 98% and correctly identified 39 out of 40 negative samples. For the VZV virus, the in‐house method identified all 20 positive samples with a sensitivity of 100%, and its specificity in negative samples was 97%, correctly identifying 68 out of 70 negative samples. Also, for EBV, the in‐house method showed 100% sensitivity in detecting 20 positive samples and 95% specificity in detecting 67 out of 70 negative samples.

No cross‐reactivity was observed during testing with other viral DNA (CMV, HHV‐6, HHV‐7, and HHV‐8) and also bacterial DNA (*Staphylococcus aureus* and *Escherichia coli*), confirming the high analytical specificity of the in‐house assay.

Overall, these results indicate the high sensitivity, specificity, and efficiency of the in‐house method in detecting positive and negative samples for HSV, VZV, and EBV viruses compared to the Altona Diagnostics reference kits (RealStar PCR Kit).

### 3.2. Comparison of Sensitivity and Specificity

Comparison of sensitivity and specificity between the in‐house method and the reference kits in detecting HSV, VZV, and EBV viruses showed similar performance of these methods. The in‐house method with 100% sensitivity and over 95% specificity provides similar results to the reference kits (Table 2). These findings indicate that the in‐house method is fully consistent with the reference kits in terms of sensitivity and specificity and has high accuracy and efficiency in detecting the aforementioned viruses.

**Table 2:** Performance of the in‐house multiplex real‐time PCR assay compared to the Altona RealStar reference method.

**Virus name**	**Number of positive samples**	**Number of negative samples**	**Sensitivity of in-house method**	**Specificity of in-house method**
HSV	50	40	100%	98%
VZV	20	70	100%	97%
EBV	20	70	100%	95%

*Note:* Sensitivity and specificity were calculated for the in‐house assay using the Altona RealStar PCR Kits as the reference standard.

### 3.3. LOD

To evaluate the LOD, serial dilutions of quantified plasmid DNA standards for HSV, VZV, and EBV were prepared. Results demonstrated that both the in‐house method and the reference Altona RealStar PCR Kits could detect these viruses at low concentrations with 100% sensitivity. The In‐house method achieved an LOD of 6.25 copies/mL for HSV and 25 copies/mL for both VZV and EBV, while the Altona RealStar PCR Kits showed an LOD of 50 copies/mL for all three viruses, as specified by the manufacturer and confirmed in our experimental validation. These results highlight the higher analytical sensitivity of the in‐house method, especially for detecting low viral loads.

### 3.4. Precision of the In‐House Method

The precision of the in‐house assay and the reference kits for HSV, VZV, and EBV detection was evaluated according to CLSI EP05‐A3 guidelines. As shown in Table 3 (intra‐assay precision) and Table 4 (interassay precision), the in‐house assay demonstrated consistently lower SD and CV values than the reference kits in both intra‐assay (repeatability) and interassay (intermediate precision) assessments. For intra‐assay precision, SD and CV values were 0.1% and 1.5% for HSV, 0.2% and 2.3% for VZV, and 0.3% and 3.7% for EBV. In the interassay assessment, the SD and CV values were 0.12% and 1.8% for HSV, 0.22% and 2.6% for VZV, and 0.33% and 3.9% for EBV. These results confirm the high precision of the in‐house assay in detecting all three viruses, both within the same analytical run and across different days.

**Table 3:** Intra‐assay precision results for the in‐house assay and reference kits in HSV, VZV, and EBV detection.

**Virus name**	**Positive samples**	**Negative samples**	**SD (in-house)**	**SD (reference kits)**	**Mean Ct (In-house)**	**Mean Ct (reference kits)**	**CV% (in-house)**	**CV% (reference kits)**
HSV	50	40	0.1	0.2	25.6	25.8	1.5%	2.1%
VZV	20	70	0.2	0.3	27.4	27.6	2.3%	3.0%
EBV	20	70	0.3	0.4	28.5	28.7	3.7%	4.5%

**Table 4:** Interassay precision results for the in‐house assay and reference kits in HSV, VZV, and EBV detection.

**Virus name**	**Positive samples**	**Negative samples**	**SD (in-house)**	**SD (reference kits)**	**Mean Ct (in-house)**	**Mean Ct (reference kits)**	**CV% (in-house)**	**CV% (reference kits)**
HSV	50	40	0.12	0.21	25.6	25.8	1.8	2.3
VZV	20	70	0.22	0.31	27.4	27.6	2.6	3.1
EBV	20	70	0.33	0.42	28.5	28.7	3.9	4.6

### 3.5. Bland–Altman Analysis

The Bland–Altman analysis was performed to assess the level of agreement between the in‐house method and the reference kits. The results, summarized in Table 5 and depicted in Figure 1, show that the mean difference (bias) for HSV and EBV was 1.35 Ct and 1.75 Ct, respectively, indicating the average difference in Ct values between the in‐house assay and the reference method. For VZV, a slightly negative mean difference was observed; however, this difference remained within an acceptable range. Importantly, no proportional bias was detected across the Ct range, indicating that assay performance was independent of Ct values and, consequently, independent of viral load. Overall, the findings demonstrate a high level of agreement between the in‐house method and the reference kits for detecting HSV, VZV, and EBV.

**Table 5:** Bland–Altman analysis for HSV, VZV, and EBV detection.

**Virus name**	**Mean difference**	**95% confidence interval (CI)**	**Lower limit**	**Upper limit**
HSV	1.35	−0.23–2.92	−9.51	12.21
VZV	−3.29	−5.20–−1.37	−11.29	4.72
EBV	1.75	0.47–3.04	−3.63	7.13

Figure 1(a–c) Bland–Altman plots for HSV, VZV, and EBV detection.

**Figure:**
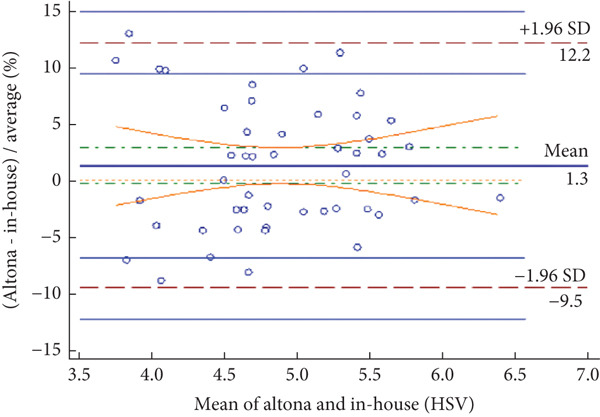
(a)

**Figure:**
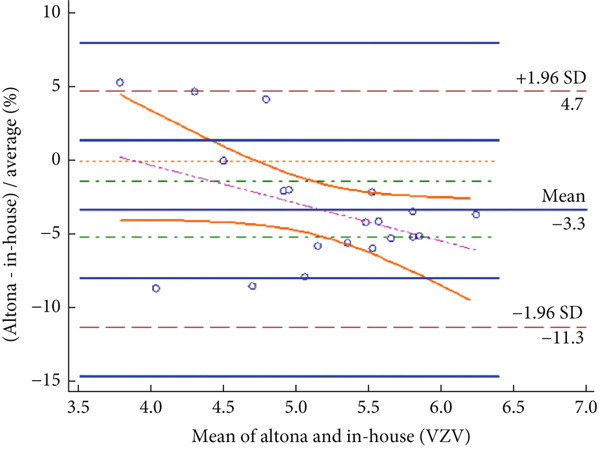
(b)

**Figure:**
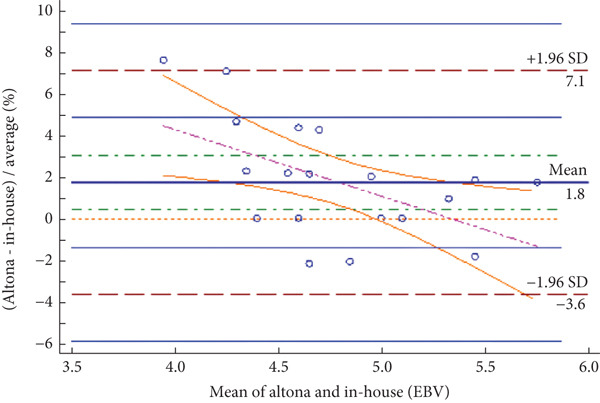
(c)

Figure 1 presents the Bland–Altman plots comparing the in‐house multiplex real‐time PCR method with the reference Altona Diagnostics RealStar PCR Kit for detecting HSV (Figure 1a), VZV (Figure 1b), and EBV (Figure 1c). The mean difference for HSV was 1.35 (95% CI: −0.23–2.92), for VZV was −3.29 (95% CI: −5.20–−1.37), and for EBV was 1.75 (95% CI: 0.47–3.04). The dashed lines represent the upper and lower limits of agreement (± 1.96 SD): for HSV (12.21, −9.51), for VZV (4.72, −11.29), and for EBV (7.13, −3.63). These plots demonstrate strong concordance between the two methods, with most values falling within clinically acceptable limits (≤ ± 2 Ct). For VZV, the mean difference slightly exceeded this threshold; however, it remained within an acceptable analytical range and did not affect the clinical outcome interpretation.

### 3.6. Linear Regression Analysis

Linear regression analysis was performed using Ct values obtained from serial dilutions of quantified plasmid standards containing the respective viral targets. These plasmid‐based standards were used solely to evaluate analytical performance and to establish the relationship between Ct values and input copy number. The coefficient of determination (*R* ^2^) for HSV, VZV, and EBV was 0.82, 0.93, and 0.94, respectively (Table 6 and Figure 2). These *R* ^2^ values indicate a strong analytical correlation of Ct values between the in‐house assay and the Altona RealStar kit when tested with plasmid‐derived standards. This analysis reflects only the analytical relationship of the assays and does not represent quantitative viral load determination in clinical samples.

**Table 6:** The results of linear regression of in‐house method and commercial kits for HSV, VZV, and EBV detection.

**Virus name**	**Coefficient of determination (** **R** ^2^ **)**	**Regression equation**
HSV	0.82	*y* = 0.8951*x* + 0.4499
VZV	0.93	*y* = 1.1213*x* − 0.4339
EBV	0.94	*y* = 1.1033*x* − 0.5765

Figure 2(a–c) Linear regression analysis for HSV, VZV, and EBV detection.

**Figure:**
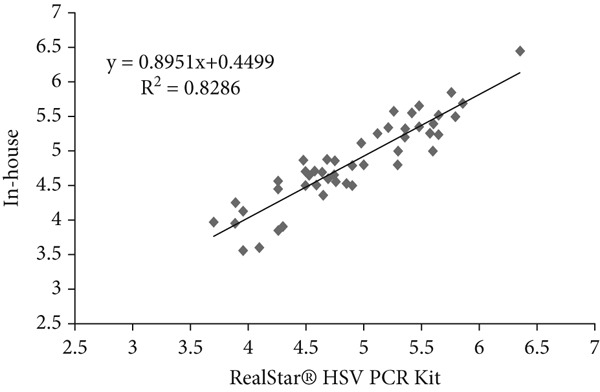
(a)

**Figure:**
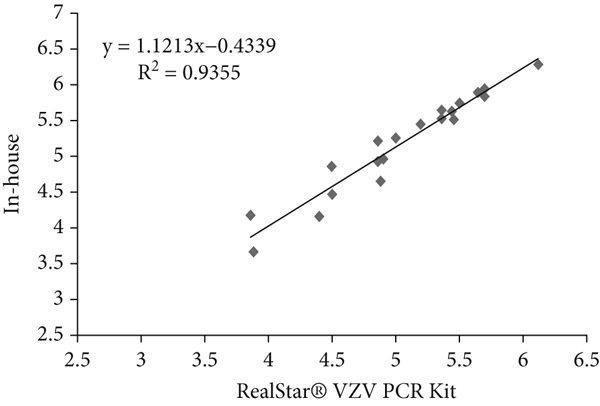
(b)

**Figure:**
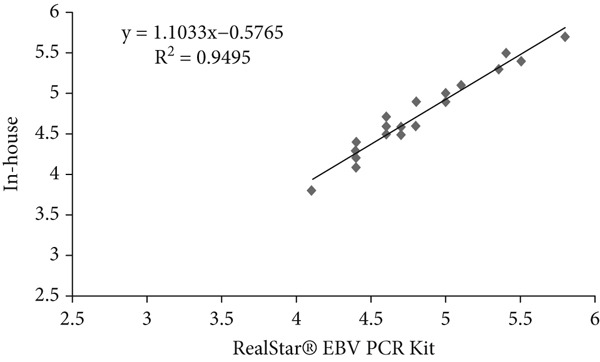
(c)

Scatter plots illustrating linear regression analysis comparing the in‐house multiplex real‐time PCR method with the Altona Diagnostics RealStar PCR Kit for detection of (Figure 1a) HSV, (Figure 1b) VZV, and (Figure 1c) EBV. The *x*‐axis represents the results from the reference kit, while the *y*‐axis shows the results from the in‐house method. These values indicate a strong analytical correlation between the Ct values obtained from the in‐house assay and those from the reference kit. This correlation reflects the consistency of Ct‐based performance between the two assays and should not be interpreted as a measure of qualitative virus detection.

## 4. Discussion

In modern medicine, accurate and rapid detection of viral infections in transplant patients and immunocompromised individuals is one of the significant challenges [12]. Due to their suppressed immune systems, these patients are at a higher risk of opportunistic infections, particularly with HSV, VZV, and EBV [13]. In such cases, prompt and simultaneous detection of these viruses plays a critical role in preventing disease progression and managing treatment strategies. Multiplex real‐time PCR methods are widely used as the standard assays for viral detection due to their high sensitivity and specificity [14].

This study evaluated the performance of an in‐house multiplex real‐time PCR method for simultaneous detection of HSV, VZV, and EBV and compared the results with those of the commercial Altona Diagnostics RealStar PCR Kit. The findings revealed that the in‐house method demonstrates high sensitivity and specificity. In fact, the method achieved 100% sensitivity for all three viruses and over 95% specificity, highlighting its high quality in the detection of target viruses.

Cross‐reactivity was also thoroughly evaluated in this study. In silico BLAST searches and in vitro cross‐reactivity testing confirmed that the primers and probes designed for this method were highly specific, with no significant homology to genomes of closely related viruses, such as CMV, HHV‐6, HHV‐7, and HHV‐8. These viruses, all members of the Herpesviridae family, share significant genetic similarities with HSV, VZV, and EBV, making them important to test for potential cross‐reactivity. This specificity ensures that the in‐house method can reliably distinguish between target viruses and nontarget organisms, preventing false‐positive results. Studies like Franco et al. have emphasized the importance of addressing cross‐reactivity in PCR methods to improve diagnostic accuracy and reduce the risk of misidentification [15].

One notable advantage of this method is its ability to detect viruses in samples with low viral loads. According to the study results, the in‐house method had an LOD of 6.25 copies/mL for HSV and 25 copies/mL for both VZV and EBV, whereas the LOD for the reference kit was 50 copies/mL. These values indicate that the in‐house method has a higher capability to detect viruses at lower concentrations, making it well suited for clinical environments that require precision, sensitivity, and specificity.

Previous studies have also emphasized the importance of sensitivity in detecting viruses at low concentrations. For instance, Rodriguez et al. reported challenges with certain commercial kits in identifying low‐viral‐load samples [16]. Our findings demonstrate that the in‐house method effectively addresses this issue, showing high analytical sensitivity in detecting HSV, VZV, and EBV. This high sensitivity reflects the assay’s ability to detect true positive cases even at low target concentrations, thereby ensuring its clinical applicability in transplant patient monitoring.

The results also demonstrated that the in‐house method provides high analytical precision across both repeatability and intermediate precision assessments. Specifically, the intra‐assay (repeatability) CV and SD values were 1.5% and 0.10% for HSV, 2.3% and 0.20% for VZV, and 3.7% and 0.30% for EBV. In the interassay (intermediate precision) evaluation, CV and SD values were 1.8% and 0.12% for HSV, 2.6% and 0.22% for VZV, and 3.9% and 0.33% for EBV. These consistently low values across both intra‐ and interassay analyses highlight the assay’s robust performance under both within‐run and day‐to‐day conditions. Importantly, when compared with the Altona Diagnostics RealStar PCR Kit, the in‐house assay consistently showed lower CV and SD values, confirming its superior analytical precision in repetition.

Similar findings were reported by Jacomasso et al., who highlighted the significance of reproducibility in molecular diagnostic methods. Their study showed that methods with lower CV and SD values are more suitable for clinical environments requiring accurate and frequent diagnostics [17]. Consistent with these findings, our results support the in‐house method as a clinically applicable diagnostic option for clinical laboratories.

Advanced statistical analyses, including Bland–Altman plots and linear regression, validated the accuracy and agreement of the in‐house method with the Altona Diagnostics RealStar PCR Kit. The results showed a mean difference of 1.35 (95% CI: −0.23–2.92) for HSV, −3.29 (95% CI: −5.20–−1.37) for VZV, and 1.75 (95% CI: 0.47–3.04) for EBV. These findings demonstrate the high accuracy of the in‐house method and approve its ability to provide results close to actual viral concentrations.

Linear regression analysis also confirmed a significant correlation (*R* ^2^ = 0.89) between the results of the in‐house method and actual viral concentrations, as validated against the Altona Diagnostics RealStar PCR Kit. Linear regression analysis confirmed a significant correlation between the Ct values obtained from the in‐house method and those from the reference kit, reflecting the consistency of analytical performance between the two assays. This strong correlation aligns with findings from Yip et al., who reported an *R* ^2^ value of 0.926 in their research [18]. Such alignment further validates the reliability of our results and highlights the potential of our in‐house method for providing precise and clinically applicable diagnostic data.

An important advantage of the in‐house method is its accessibility and adaptability for laboratories, particularly in resource‐limited settings. By reducing dependency on commercial kits, laboratories may benefit from increased flexibility in diagnostic capacity. Moreover, the method enables simultaneous detection of HSV, VZV, and EBV in a single PCR reaction. This multiplexing feature saves time and laboratory resources, which is especially valuable in clinical settings where rapid and efficient diagnostics are critical for timely patient management [19].

Despite these promising results, some limitations must be acknowledged. One limitation is the relatively small sample size used in this study. To confirm these findings and enhance their validity, larger and more diverse sample sizes are needed in future research. Additionally, this study focused solely on three viruses (HSV, VZV, and EBV). Future investigations could expand the scope to include other related viruses, such as CMV and other herpesviruses.

## 5. Conclusion

This study demonstrates that the in‐house multiplex real‐time PCR method is a practical and clinically applicable diagnostic tool for the simultaneous detection of HSV, VZV, and EBV in clinical laboratories. This approach not only improves diagnostic processes but also enhances the speed of diagnosis and supports timely clinical decision‐making. Leveraging local technologies in developing this method can positively impact healthcare access in various communities, ultimately improving diagnostic outcomes for patients.

## Ethics Statement

The study was approved and under the supervision of the Biomedical Ethics Committee of Iran (ethics code: IR.IAU.KAU.REC.1403.006).

## Disclosure

All the authors read and approved the final version of the manuscript.

## Conflicts of Interest

The authors declare no conflicts of interest.

## Author Contributions

**Reyhaneh Kalhor:** performing the project, collecting data, writing – original draft. **Mahdi Paryan:** supervision, methodology, reviewing and editing the manuscript. **Sirous Naeimi:** supervision and methodology. **Hourieh Kalhor:** conceptualization. **Hassan Noorbazargan:** data curation. **Samira Mohammadi-Yeganeh:** validation, reviewing and editing.

## Funding

This work was funded by the Iran’s Islamic Azad University, Kazeroon Branch, Kazeroon, Iran.

## Data Availability

The data that support the findings of this study are available from the corresponding author upon reasonable request.
